# Headmistresses on Nursing as a Profession

**Published:** 1921-04-02

**Authors:** 


### Headmistresses on Nursing as a Profession.

At an interview held at the College of Nursing Headquarters between representatives of the Headmistresses' Association and the Council of the College, the difficulty in recruiting educated girls as candidates for the nursing profession was fully discussed, and the points of view of the girls and their guardians as the headmistresses find them were explained.

Six Drawbacks.

Amongst drawbacks to entrance into the profession of nursing the following were put forward: (1) The low increment to the qualified nurse's salary; (2) the somewhat rigid discipline; (3) alleged lack of consideration sometimes shown by doctors to nurses; (4) absence of any superannuation scheme; (5) absence of any standard of general education for admission to the training-schools; (6) the long interval between leaving school and beginning the training for a nurse.

Charges, Changes, and Defence.

Taking the above "drawbacks" in order—(1) It was suggested that as the working years of a nurse are less than those of other women the annual increment should be not less than £10, and that the minimum salary of a qualified nurse should be £85, rising to £200; (2) probationers usually come in contact with the Matron when there is occasion for rebuke, which tends to emphasise the strictness of the discipline; (3) it was pointed out by the representatives of nurses that though there might be isolated cases of lack of consideration, as a general rule there was absolute loyalty on the part of nurses towards doctors; (4) it was urged that until nursing is recognised as National Service and a superannuation scheme inaugurated, as in the teaching profession, nursing as a profession will be at a disadvantage; (5) it was felt that the institution of a standard examination for admission to training, such as the "First" examination of the Board of Education, would raise the status of the whole profession. A candidate failing to reach this standard should not necessarily be excluded from entering the profession if her school record showed a good general education. It was suggested that the College of Nursing should institute its own examination for candidates educated privately; (6) it was suggested that the establishment of Central Preliminary Schools to include domestic science would help to bridge over the period between leaving school and entering the hospital training-school.

On the question of remuneration, it was pointed out, in answer to criticisms from the Headmistresses' Association, that the emoluments of board, lodging, laundry, and medical and nursing attendance provided for nurses in hospital must be taken into consideration. These should be calculated at £120 to £150 annually.

Closer Co-operation.

At the close of the conference representatives of the Headmistresses' Association were invited to visit some of the nurse training-schools, and see the conditions under which the nurses worked. This invitation was most readily accepted. It was felt that the result of the conference must be a closer co-operation between the schools and the nursing profession.

[The effect of some of the drawbacks here mentioned is discussed in the article on p. 17, "The Pay of the Nurse."—Ed. The Hospital.]

